# Update on the Surgical Trial in Lobar Intracerebral Haemorrhage (STICH II): statistical analysis plan

**DOI:** 10.1186/1745-6215-13-222

**Published:** 2012-11-21

**Authors:** Barbara A Gregson, Gordon D Murray, Patrick M Mitchell, Elise N Rowan, Anil R Gholkar, A David Mendelow

**Affiliations:** 1 STICH Office, Neurosurgical Trials Unit, Newcastle University, 3-4 Claremont Terrace, Newcastle upon Tyne, NE2 4AE, UK; 2 Centre for Population Health Sciences, University of Edinburgh Medical School, Teviot Place, Edinburgh, EH8 9AG, UK; 3 Regional Neurosciences Centre, The Newcastle upon Tyne Hospitals NHS Foundation Trust, Royal Victoria Infirmary, Queen Victoria Road, Newcastle upon Tyne, NE1 4LP, UK

## Abstract

**Background:**

Previous studies had suggested that the outcome for patients with spontaneous lobar intracerebral haemorrhage (ICH) and no intraventricular haemorrhage (IVH) might be improved with early evacuation of the haematoma. The Surgical Trial in Lobar Intracerebral Haemorrhage (STICH II) set out to establish whether a policy of earlier surgical evacuation of the haematoma in selected patients with spontaneous lobar ICH would improve outcome compared to a policy of initial conservative treatment. It is an international, multi-centre, prospective randomised parallel group trial of early surgery in patients with spontaneous lobar ICH. Outcome is measured at six months via a postal questionnaire.

**Results:**

Recruitment to the study began on 27 November 2006 and closed on 15 August 2012 by which time 601 patients had been recruited. The protocol was published in *Trials* (http://www.trialsjournal.com/content/12/1/124/). This update presents the analysis plan for the study without reference to the unblinded data. The trial data will not be unblinded until after follow-up is completed in early 2013. The main trial results will be presented in spring 2013 with the aim to publish in a peer-reviewed journal at the same time.

**Conclusion:**

The data from the trial will provide evidence on the benefits and risks of early surgery in patients with lobar ICH.

**Trial registration:**

ISRCTN: ISRCTN22153967

## Update

### Introduction

The Surgical Trial in Lobar Intracerebral Haemorrhage (STICH II) aims to establish whether a policy of earlier surgical evacuation of the haematoma in selected patients with spontaneous lobar intracerebral haemorrhage (ICH) will improve outcome compared to a policy of initial conservative treatment. The trial will also help to better define the indications for early surgery.

This is an international multi-centre parallel group trial with patients randomised to receive either ‘early surgery’ (ES) or ‘initial conservative treatment’ (ICT). Outcome is measured at six months by postal questionnaire to the patients.

Eligible patients have evidence of a spontaneous lobar ICH on computed tomography (CT) scan (1 cm or less from the cortical surface of the brain) with a volume of between 10 and 100 ml, are within 48 hours of ictus, have a best motor score on the Glasgow Coma Score (GCS) of five or six and best eye score of two or more. They are not eligible if there is clear evidence that the haemorrhage is due to an aneurysm or angiographically proven arteriovenous malformation, that it is secondary to tumour or trauma, involves the basal ganglia, thalamic, cerebellar or brainstem regions, or if there is any intraventricular blood. Patients should also not have any severe pre-existing physical or mental disability or severe co-morbidity that might interfere with assessment of outcome. They should be able to have surgery within 12 hours if randomised to that group.

All patients who are randomised and have outcome measured will be included in the analysis. Patients who withdraw consent from the study will not be included. The primary analysis will be ‘intention to treat’ with data analysed according to the treatment group to which the patient was randomised. This update to the published protocol [1] provides details of the statistical analysis plan for STICH II.

### Background to the statistical analysis of STICH II

#### Results from similar trials

**Figure 1:**
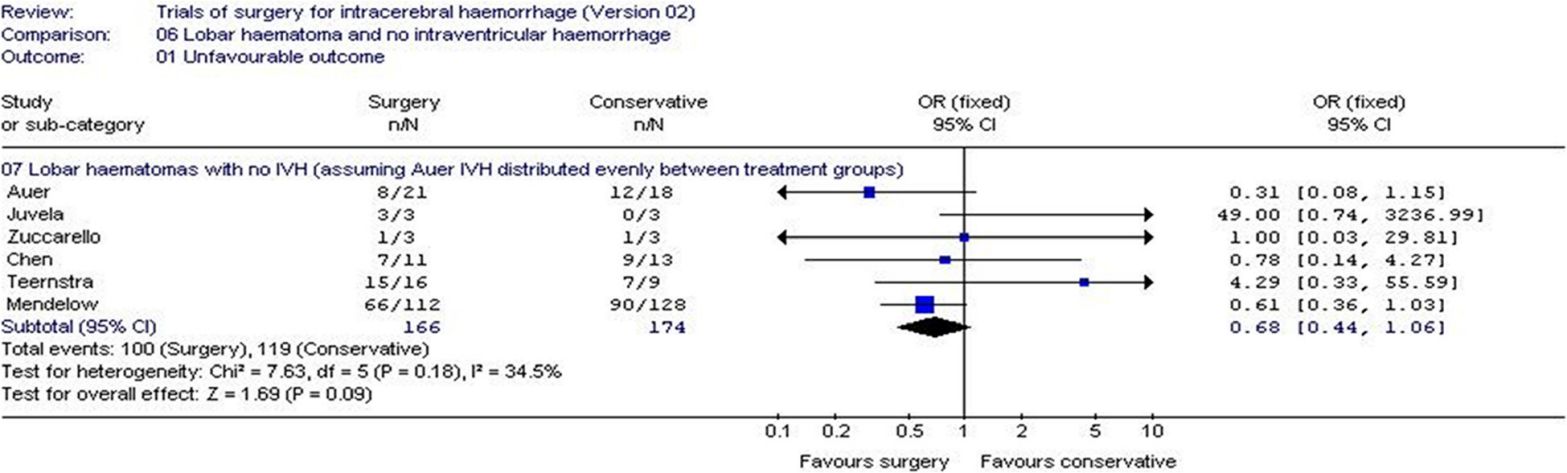
**Individual patient data meta-analysis of surgery versus conservative treatment for the subgroup of patients with lobar ICH and no IVH.** Reproduced from Gregson et al. [2].

Some early work was reported in the protocol from a meta-analysis of individual patient data from previous studies. This work has now been extended and published [2]. In particular, the analysis of patients with only lobar haematomas and no intraventricular haemorrhage (IVH) using unfavourable outcome defined as dead, severely disabled or independent inside the home shows a tendency to favour surgery (Figure 1). This analysis is dominated by the Surgical Trial in Intracerebral Haemorrhage (STICH) [3], which was the motivation for undertaking STICH II.

#### Further detailed analysis of data from the STICH study

In order to plan data analysis of STICH II, it is useful to examine the data collected in the first STICH study [3] for cases that would have been eligible for STICH II under the present inclusion criteria.

In particular, the haematoma should be located in the lobar region with no haematoma in the basal ganglia or thalamus or in the ventricles and no more than 1 cm from the cortex surface. The size of the haematoma should be between 10 and 100 ml. The patient’s GCS motor component at randomisation should be five or six and the eye component two or more. The patient should be within 48 hours of ictus.

For values of the size and location of blood in the brain, we have available to us measures reported by the local investigator at the time of randomisation and also the assessments made by the central examiners from the measurements made on the randomisation CT scan supplied to us. Since it is the local investigator who decides whether a patient is eligible or not, for this analysis the measurements reported on the randomisation form will be used. However, presence of IVH was not reported at randomisation so that information will be taken from the central assessment.

This selection provides 157 patients who had all the inclusion criteria (78 randomised to ES and 79 to ICT). Outcome at six months is available for 147 of these patients (74 ES, 73 ICT). Of the ten lost patients, no follow-up at all was obtained for six patients and four patients were known to be alive at six months but died before follow-up could be obtained so disability at six months is unknown. There were a few discrepancies between the randomisation assessment and the central assessment of haematoma characteristics. The depth from the cortical surface exceeded 1 cm in four cases (maximum 1.8 cm). The volume was less than 10 ml in one case (8.2 ml) and more than 100 ml in four cases (101.2, 101.7, 103.2 and 137.3).

Dichotomising the five-point Glasgow Outcome Scale (GOS) at the usual point (good recovery (GR), moderate disability (MD) versus severe disability (SD), vegetative (V), dead (D)) gave 37% favourable outcome overall with 45% in the ES group and 30% in the ICT group (Fisher’s exact *P* = 0.089, Pearson’s chi-squared = 0.070). Using the prognostic-based outcome reported in the *Lancet* paper [3] gives 41% favourable outcome overall (49% in the ES group and 33% in the ICT group).

For the 147 patients with outcome assessment, the two treatment groups were well matched on the usual predictive variables of age <65 (42% ES and 42% ICT) and volume >50 ml (31% ES and 32% ICT), but for GCS 13 to 15 (70% ES and 63% ICT) and whether the patient was randomised within 12 hours (24% ES and 36% ICT), there was evidence of some differences.

In the surgery group, 52 patients (70%) had surgery within 12 hours, 2 patients did not receive surgery and 8 had surgery after 24 hours (26 to 49). In the initial conservative group, 25 (34%) had surgery, 8 within 12 hours of randomisation and 13 had surgery after 24 hours.

**Figure 2:**
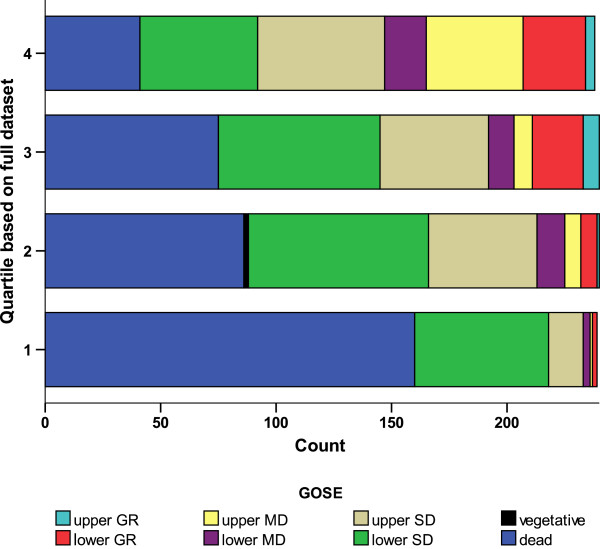
Distribution of outcome by quartile of prognostic index for all patients in STICH.

Dividing the prognostic score (10 x GCS – age – 0.64 x volume) according to the values of the quartiles and comparing that with the eight-point GOS at six months gives the result shown in Figure 2 for all patients in STICH. (Values of the quartiles are −3.66, 27.672, 53.78)

**Figure 3:**
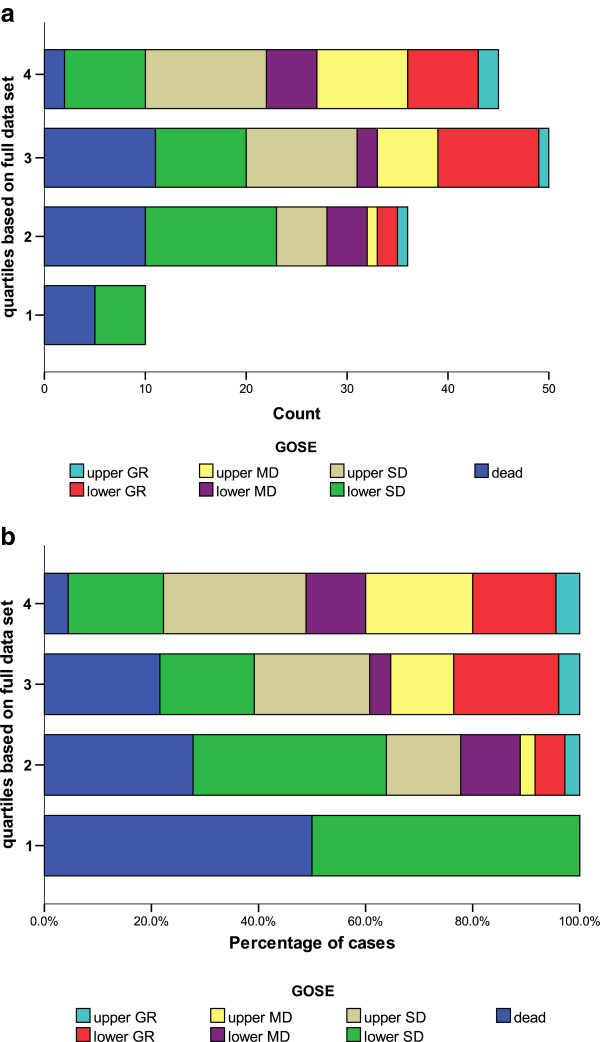
**Distribution of outcome by quartile of prognostic index for patients in STICH that would have been eligible for STICH II.(a)** Count of cases. **(b)** Percentage of cases.

Figure 3 shows the plot of eight-point GOS for only those STICH patients who would have fulfilled the criteria for STICH II plotted against the baseline risk score quartiles (as calculated from all the patients in STICH). Figure 3a shows the graph for number of respondents and Figure 3b for the percentage of respondents.

#### Sample size in STICH II

In the protocol for STICH II, it is stated that with a 37% favourable outcome from conservative treatment a sample size of 566 would be required to show a 12% benefit from surgery (2*P* <0.05) with 80% power. A sample size of 600 was therefore proposed to allow for withdrawals and crossovers.

#### Randomisation procedures in STICH II

Patients are allocated according to a minimisation algorithm based on age (three bands: <60, 60 to 69, 70+) and neurological deficit in the worst affected arm or leg (three bands: normal, weak, paralysed) with a random component such that there is a 20% chance of the allocation being reversed. Patients are stratified by country group and planned operation (two bands: craniotomy, other). Sites are grouped by country (if we expected many sites within a country to be included) or a country group (if we expected only one site in the country). Country groups were defined, as they arose, according to geographic location.

#### Analysis statement in protocol for STICH II

The analysis statement in the protocol says: analysis will be on an ‘intention to treat’ basis. The primary analysis will be a simple categorical frequency comparison using the chi-squared test for prognosis-based [4,5] favourable and unfavourable outcomes at six months. Patients with a good prognosis will be categorised as having a favourable outcome if they achieve GR or MD on the GOS. Patients with a poor prognosis will be categorised as having a favourable outcome if they achieve GR, MD or upper SD on the Glasgow Outcome Scale-Extended (GOSE). Logistic regression analysis will be undertaken to adjust for covariates. Secondary outcomes will also be analysed using the prognosis-based method as specified in STICH [3].

#### Statistics that will be reported

For categorical variables, the number and percentage in each group will be reported. Percentages will be reported to no decimal places. For continuous variables the median, quartiles, maximum and minimum will be reported. Where *P* values are reported these will be to three decimal places or at *P* <0.0001. The presence of missing data will be reported.

### Methods for statistical analyses to be reported in STICH II

#### Evaluation of demographics and baseline characteristics

Baseline characteristics will be reported separated by treatment. The first table will include gender, age, pre-ICH mobility, pre-ICH Rankin, time between ictus and randomisation, GCS, neurological deficit in worst arm, worst leg, and worst limb, speech, whether receiving any anticoagulation or thrombolytic treatment prior to ictus, past medical history and prognostic score. Prognostic score will be derived as reported in STICH (10 x GCS – age (years) – 0.64 x volume (ml)) with a cut point of 27.672 to differentiate between patients with a good prognosis (score greater than cut point) and those with a poor prognosis.

The second table will include details of site of haematoma, volume and depth from the site assessments plus those and other measures from the central assessment.

#### Evaluation of treatment compliance and exposure

Crossovers will be reported. The third table will include details of surgery for both groups including time from ictus to surgery, and number and percentage operated within 12 hours of ictus, time from randomisation to surgery, method, any additional procedures, and status prior to evacuation, whether sedated, GCS, and neurological status of worst arm, leg and speech. Reasons for crossover will be reported. Amount of blood removed during operation will be reported (as assessed by differences in blood volume on randomisation scan and on the five-day CT scan).

#### Outcome analysis

Outcomes will be reported as odds ratios with 95% confidence interval reported to two decimal places and *P* values to three decimal places. Absolute benefits with 95% confidence intervals to one decimal place will also be reported.

The primary outcome analysis will be a simple categorical frequency comparison using the chi-squared test for prognosis-based favourable and unfavourable outcomes on the eight-point GOS at six months. GOS will be computed from the answers to the 14 questions as per the paper of Wilson, Edwards et al. [6] Where a response to a question is missing, the assessment will be made using the responses to the other questions. The allocation of outcome assessment will be made prior to unblinding the treatment assignment.

Logistic regression analysis will be undertaken to adjust for covariates age, GCS, volume of haematoma and neurological deficit.

Six-month outcome assessment may actually be made any time after five months. Where it is very late, the respondents are asked to answer the questions for an assessment of the capabilities of the patient at the six-month point. If a patient dies after six months but prior to the six-month outcome being completed and it is impossible to obtain an assessment of the patient’s abilities at six months, the patient will be excluded from analysis of GOS.

Secondary outcome analysis will include Kaplan-Meier survival curve with log-rank test, mortality at six months, a prognosis-based modified Rankin score, reporting of the eight-point GOS, Rankin and EuroQol broken down by randomised treatment group.

#### Sensitivity analyses

An analysis based on a proportional odds model will be undertaken as a sensitivity analysis. In addition, sensitivity analyses will be undertaken based on imputation of missing outcome data.

#### Subgroup analysis

Odds ratios and 95% confidence intervals will be reported for the following subgroups: age (two bands using median split: <65, >=65), volume of haematoma (using median split <= 35 ml, >35 ml), GCS (8 to 12, 13 to 15), time from ictus to randomisation (using median split <21 hours, >=21 hours), severity of neurological deficit - worse limb (normal, weak, paralysed), planned method of haematoma removal (craniotomy, other). Interaction tests will be undertaken and relevant *P* values will be reported.

In addition, odds ratios will be reported by country group and by whether the patients had been receiving anticoagulant treatment prior to the haemorrhage.

#### Subsidiary analyses

Subsidiary analyses will be conducted although we recognise that any analysis by treatment received will be biased and extremely difficult to interpret. They will not be reported in the initial paper. Since delayed surgery is permitted by the protocol, we will carry out two different per protocol analyses excluding patients in the ICT group who have surgery within a) 12 hours or within b) 48 hours:

a) Per protocol (12 hours) - only patients who received the allocated treatment within 12 hours will be included in this analysis. ES patients will have received surgery within 12 hours or died within 12 hours. ICT patients will not have received surgery within 12 hours.

b) Per protocol (surgery versus conservative) - only patients who received the allocated treatment will be included in this analysis. ES patients will have received surgery within 12 hours. ICT patients will not have received surgery within 48 hours.

#### Other analyses

In separate papers, the analysis of the CT scans will be reported. Each CT scan will be read by two independent blinded readers: a neuroradiologist and a neurosurgeon. They are not informed as to whether the scan is a diagnostic scan or a five-day scan and the scans are coded such that they cannot link two scans for one patient. Although the readers will be able to identify if an operation has been undertaken, they will not know which group the patient was randomised to. They will report the location and size of the haematoma, the presence of blood in the ventricles, any midline shift or evidence of other pathology. The inter-rater reliability will be reported. Where there is evidence of disagreement, a third reader will assess the scan. Percentage change in volume of haematoma between the two scans will be compared with outcome according to volume of haematoma on the diagnostic scan.

### Other issues

1. *Incorrect randomisation*. There have been a couple of incidences when there have been problems with randomisation. On several occasions, the database manager was given information that the patient had been randomised to one treatment but the investigator had been told the other treatment by the randomisation service. If the investigator acted on the information from the randomisation service this was correct. If the investigator acted on information from the database manager then this was taken as correct and the randomisation service was asked to edit their database. This was a problem created by the randomisation service in Aberdeen, which was identified after a six-month period and affected three patients.

2. *Treatment allocation not heard*. On seven occasions the investigator reported that they did not hear the treatment during the phone call (the phone cut out). He/she then went through the system again resulting in the patient being randomised twice. The investigator then used the information from the second call. Sometimes this was the same and sometimes different. If this happened during a working day the database manager immediately contacted the site and asked them to follow the initial allocation. If it was not possible to make this contact immediately then the second allocation was used. In both cases, the randomisation service was contacted to ensure the correct minimisation algorithm was maintained. This occurrence was not related to site.

3. *Missing data*. Missing data are rare because sites are contacted to ensure that all data collected by two weeks is available. Sometimes a randomisation CT was not obtained because the patient took it home with them before it was copied or it had been done at an outlying hospital and a copy could not be obtained. For these cases, it will not be possible to estimate change in volume of the haematoma by treatment as estimated by blinded raters. Missing outcome information has been addressed above.

4. *Protocol variations*. Some patients may have been recruited who were later found to have been ineligible (central assessment of the CT scan may identify the haematoma as being outside the eligible volume or there might be evidence of blood in the ventricles or of a bleed involving deeper structures, or caused by other mechanisms). These will be detailed but included in all analyses as if appropriately recruited.

### Discussion and timetable

Patients were recruited to the trial between 27 November 2006 and 15 August 2012. In total, 601 patients were recruited. Follow-up of recruited patients will continue through 2012 and into 2013. The trial data will be unblinded in early 2013 once data collection is complete and the dataset is closed. The main trial results will be presented in the spring of 2013. The aim will be to publish the results at the same time or soon after.

### List of all registered recruiting collaborating centres

ARMENIA - Yerevan State Medical University, Dr Ruben V Fanarjyan

AUSTRALIA - Royal Melbourne Hospital, Prof Stephen Davis

AUSTRIA - Rudolfstiftung Hospital, Vienna, Dr Günther Kleinpeter

Medical University of Vienna, Prof Andreas Gruber

CANADA - University of Alberta Hospital, Edmonton, Dr Max Findlay

CHINA - Tianjin Medical University General Hospital, Prof Shuyuan Yue

Beijing Tiantan Hospital, Dr Yuanli Zhao

Huashan Hospital, Fudan University, Shanghai, Prof Dr Ying Mao

CZECH REPUBLIC - Central Military Hospital, Prague, Prof Vladimir Benes

Fakultni Nemocnice, Ostrava, Dr Tomas Krejci

University Hospital, Brno, Prof Martin Smrcka

St. Anne's University Hospital, Brno, Prof Dr Pavel Cejpak

Fakultni Nemocnice, Olomouc, Prof Miroslav Vaverka

Regional Hospital, Liberec, Dr Pavel Buchvald

EGYPT - Mansoura International Hospital, Dr Abd-Elhafiz Shehab Eldien

Zagazig University Hospital, Dr Mohamed Barakat

Alexandria University Hospital, Prof Osama Abdelaziz

GEORGIA - State Medical University Clinic, Tbilisi, Prof Alexander Gvelesiani

GERMANY - Klinik fur Neurochirurgie, Magdeburg University, Prof Dr Raimund Firsching

Clemens Hospital, Munster, Prof Dr Abolghassem Sepehrnia

Universitatsklinikum Erlangen, Prof Dr Michael Buchfelder

Klinikum Amberg, Dr Andrea Kleindienst

Heinrich Heine University, Duesseldorf, Dr Daniel Haenggi

University Hospital Heidelberg, Dr Karl Kiening

University Clinic, Munster, Prof Walter Stummer

Klinikum Kassel, Prof Dr Wolfgang Deinsberger

Ernst Moritz Arndt University, Greifswald, Prof Dr W S Schroeder

University Schleswig-Holstein, Lubeck, Dr Georg Nowak

Helios Klinikum Berlin Buch, Prof Dr Jurgen Kiwit

Neurochirurgische Klinik, Dessau, Dr Kazimierz Sadowy

Universitatsklinikum des Saarlandes, Prof Wolf-Ingo Steudel

Dr. Horst Schmidt Kliniken, Wiesbaden, Prof Gerhard Hamman

Klinikum Saarbrucken, Winterberg, Dr S Thomas

Charite - University Medicine, Berlin, Prof Eric Juettler

Universitatsklinikum Jena, Dr Albercht Waschke

Asklepios Klinik Altona, Prof Dr Uwe Kehler

Diakonieklinikum, Jun-Stilling Hospital, Siegen, Dr Veit Braun

GREECE - Ippokration General Hospital, Aristotle University, Thessaloniki, Prof Philippos Tstitsopoulos

Evangelismos Hospital, Athens, Mr George Stranjalis

AHEPA General Hospital, Aristotle University, Thessaloniki, Dr Athanasios Spiliotopoulos

HUNGARY - Pecs University Hospital, Dr Andras Buki

Borsod County and University Teaching Hospital, Dr Jozsef Dobai

INDIA - All India Institute of Medical Sciences, New Delhi, Dr P Sarat Chandra

BGS Global Hospital, Bangalore, Dr Shailesh Rao

Christian Medical College and Hospital, Ludhiana, Dr Sarvpreet Singh Grewal

National Neurosciences Centre, Kolkata, Dr K Sridhar

Care Hospital, Visakhapatnam, Dr V P Ramana

Care Hospitals Institute of Neurological Sciences, Hyderabad, Dr T V R K Murthy

Kerala Institute of Medical Sciences, Dr Moni Vinod

Acharya Vinoba Bhave Rural Hospital, Maharashtra, Dr Anand Kakani

Kamineni Hospital, Hyderabad, Dr Subodh Raju

Amri Hospital, Dhakuria, Prof R N Bhattacharya

MM Institute of Medical Sciences and Research, Haryana, Prof Amit Agrawal

Sree Chitra Tirunal Institute for Medical Sciences and Technology, Trivandrum, Dr Suresh Nair

National Institute of Mental Health and Neuro Sciences, Bangalore, Dr B Indira Devi

ISRAEL - Rambam Hospital, Haifa, Dr Leon Levi

Sheba Medical Center, Dr Sagi Harnof

ITALY - University Sapienza, Rome, Prof Robert Delfini

JAPAN - Kanto Medical Center, NTT East Co. Tokyo, Dr Akio Morita

Hirosaki University School of Medicine, Professor Norihito Shimamura

LATVIA - Clinical University Hospital, Gailezers, Riga, Dr Kaspars Auslands

Pauls Stradins Clinical University Hospital, Riga, Prof Egils Valeinis

LITHUANIA - Klaipeda University Hospital, Prof Antanas Gvazdaitis

Kaunas University Hospital, Dr Antanas Gvazdaitis

MACEDONIA - Clinical Center, Skopje, Prof Dr Kiril Lozance

MALAYSIA - Universiti Sains Malaysia, Kubang Kieran, Prof Dr Jafri Malin

Hospital Sultanah Aminah, Johor Bahru, Dr Noor Azman Abdul Rahman

MEXICO - Instituto Nacional de Neurologia y Neurochirugia, Tlalpan, Dr Samuel Romero Vargas

Hospital Civil de Guadalajara, Dr Jose Luis Ruiz-Sandoval

MOLDOVA - National Practical and Scientific Centre of Emergency Medicine, Chisinau, Prof Stanislaw Groppa

NEPAL - B. P. Koirala Institute of Health Sciences, Dharan, Dr Yam Bahadur Roka

B & B Hospital, Gwarko, Lalitpur, Dr Krishna Sharma

NORWAY - St Olavs Hospital, Trondheim University Hospital, Dr Sozaburo Hara

PAKISTAN - Lahore General Hospital, Dr Khalid Mahmood

NW General Hospital and Research Centre, Peshawar, Prof Tariq Khan

POLAND - Poznan University of Medical Sciences, Prof Dr Stanislaw Nowak

University Medical School, Bialystok, Prof Zenon Mariak

ROMANIA - Cluj County Emergency Hospital, Cluj-Napoca, Prof Ioan Stefan Florian

Country Hospital, Timisoara, Dr Horia Ples

RUSSIA - Novosibirsk Medical University, Prof Alex Krivoshapkin

SAUDI ARABIA - King Khalid University Hospital, Riyadh, Dr Essam A Elgamal

SINGAPORE - National University Hospital, Dr Chou Ning

SOUTH AFRICA - Steve Biko Academic Hospital, Pretoria, Professor Sam Mokgokong

SOUTH KOREA - National Medical Center, Seoul, Prof Dae Hee Han

SPAIN - Hospital Universitario 'Marques de Valdecilla', Santander, Prof Alfonso Vazquez-Barquero

Hospital Universitario Virgen de las Nieves, Granada, Dr Majed J Katati

Hospital Clinico Universitario, Valladolid, Dr Rosario Sarabia

Hospital de Cruces, Bilbao, Prof Jesus Garibi Undabarra

Germans Trias I Pujol University Hospital, Barcelona, Dr Carlos Alonso

Hospital Universitario Rio Hortego, Valladolid, Dr Rosario Sarabia

University Son Espases, Majorca, Dr Javier Ibanez

University Hospital Murcia, Dr Marcelo Galarza

SRI LANKA - National Hospital of Sri Lanka, Colombo, Dr H D S Kularathne

SWEDEN - Akademiska Sjukhuset, Uppsala, Prof Per Enblad

TAIWAN - Taipei Medical University Hospital, Dr Kuo-Hsing Liao

THE NETHERLANDS - University Medical Centre, Groningen, Dr Gert-Jan Luijckx

TURKEY - University of Istanbul, Prof Orhan Barlas

UK - Ninewells Hospital, Dundee, Professor M Sam Eljamel

Radcliffe Infirmary, Oxford, Mr Richard Kerr

Hurstwood Park, Haywards Heath, Mr Giles Critchley

James Cook University Hospital, Middlesbrough, Mr Roger Strachan

The National Hospital for Neurology and Neurosurgery, London, Mr Laurence Watkins

Aberdeen Royal Infirmary, Dr Pragnesh Bhatt

Southampton University Hospital, Mr Antonio Belli, Mr Diederik Bulters

Addenbrooke's Hospital, Cambridge, Mr Peter Kirkpatrick

Newcastle General Hospital and Royal Victoria Infirmary, Newcastle, Mr John Crossman

Leeds General Infirmary, Mr Jake Timothy

Morriston Hospital, Swansea, Mr Robert Redfern

Salford Royal Infirmary, Mr Andrew King

Preston Royal Infirmary, Mr Aprajay Golash, Mr Nihal Gurusinghe

Western General Hospital, Edinburgh, Dr Rustam al-Shahi Salman

St. George's Hospital (Atkinson Morley's), London, Prof B A Bell

The Walton Centre, Liverpool, Mr Mohsen Javadpour, Mr Paul Eldridge

University Hospital of North Staffordshire, Mr Simon Shaw

Hull Royal Infirmary, Mr Shailendra Achawal

USA - Research Medical Center, Kansas, Dr Iftekar Ahmed

Hartford Hospital, Dr Inam Kureshi

Loyola University Hospital, Chicago, Dr Michael Schneck

Temple University Hospital, Philadelphia, Dr Michael Weaver

Central Illinois Neuroscience Foundation, Bloomington, Dr Keith Kattner

Georgia Neurosurgical Institute, Macon, Dr Arthur Grigorian

Albany Medical Centre, Dr John German

Mayo Clinic, Jacksonville, Dr Benjamin Eidelman

Penn State Hershey Medical Centre, Dr Kevin Cockroft

University of Louisville and Baptist Hospital East, Dr Jonathan E Hodes

University of Virginia Medical Center, Charlottesville, Dr Kenneth Liu

St. Joseph’s Candler Health System, Savannah, Dr Jay Howington

University of Illinois Medical Center at Chicago, Dr Sepidah Amin-Hanjani

Washington University School of Medicine, Dr Gregory Zipfel

## Abbreviations

CT: computed tomography; D: dead; ES: early surgery group; GCS: Glasgow Coma Score; GOS: Glasgow Outcome Scale; GOSE: Glasgow Outcome Scale - Extended (eight-point); GR: good recovery; ICH: intracerebral haemorrhage; ICT: initial conservative treatment group; IVH: intraventricular haemorrhage; MD: moderate disability; SD: severe disability; STICH: Surgical Trial in Intracerebral Haemorrhage; STICH II: Surgical Trial in Lobar Intracerebral Haemorrhage; V: vegetative.

## Competing interests

Professor A David Mendelow is a director of the Newcastle Neurosurgery Foundation Ltd and has received honoraria for attending advisory committee meetings for Stryker.

## Authors’ contributions

ADM, PMM and BAG are responsible for the conception of the study, protocol design and study coordination. GDM has provided statistical guidance throughout the study and assistance with initial study design. BAG and GDM prepared the analysis plan. ARG coordinates and provides support with CT scan acquisition and analysis and has also been involved with the development of the study. ENR has been involved with study management. All authors have been involved in the drafting of the manuscript. All authors read and approved the final manuscript.
